# Understanding communicative intentions in schizophrenia using an error analysis approach

**DOI:** 10.1038/s41537-021-00142-7

**Published:** 2021-02-26

**Authors:** Alberto Parola, Claudio Brasso, Rosalba Morese, Paola Rocca, Francesca M. Bosco

**Affiliations:** 1 Dipartimento di Psicologia, Università degli Studi di Torino, Torino, Italia; 2 Dipartimento di Neuroscienze “Rita Levi Montalcini”, Università degli Studi di Torino, Torino, Italia; 3 Institute of Public Health, Faculty of Biomedical Sciences, Università della Svizzera italiana, Lugano, Switzerland; 4 Faculty of Communication, Culture and Society, Università della Svizzera italiana, Lugano, Switzerland

**Keywords:** Schizophrenia, Human behaviour

## Abstract

Patients with schizophrenia (SCZ) have a core impairment in the communicative-pragmatic domain, characterized by severe difficulties in correctly inferring the speaker’s communicative intentions. While several studies have investigated pragmatic performance of patients with SCZ, little research has analyzed the errors committed in the comprehension of different communicative acts. The present research investigated error patterns in 24 patients with SCZ and 24 healthy controls (HC) during a task assessing the comprehension of different communicative acts, i.e., sincere, deceitful and ironic, and their relationship with the clinical features of SCZ. We used signal detection analysis to quantify participants’ ability to correctly detect the speakers’ communicative intention, i.e., sensitivity, and their tendency to wrongly perceive a communicative intention when not present, i.e., response bias. Further, we investigated the relationship between sensitivity and response bias, and the clinical features of the disorder, namely symptom severity, pharmacotherapy, and personal and social functioning. The results showed that the ability to infer the speaker’s communicative intention is impaired in SCZ, as patients exhibited lower sensitivity, compared to HC, for all the pragmatic phenomena evaluated, i.e., sincere, deceitful, and ironic communicative acts. Further, we found that the sensitivity measure for irony was related to disorganized/concrete symptoms. Moreover, patients with SCZ showed a stronger response bias for deceitful communicative acts compared to HC: when committing errors, they tended to misattribute deceitful intentions more often than sincere and ironic ones. This tendency to misattribute deceitful communicative intentions may be related to the attributional bias characterizing the disorder.

## Introduction

Pragmatics has traditionally been defined as the ability to communicate appropriately in a social context using language^1,2^. In order to understand our partners’ communicative intentions, we are often required to use inferential processes to fill the gap that occurs between the literal meaning of an utterance and the speaker’s meaning, i.e., what the speaker intends to communicate with that utterance, as for example in “Well done!” said by the speaker to ironically remark to the listener that s/he barely kicked the ball and it hit a plant pot standing on a window sill. Several studies in the literature have demonstrated that patients with schizophrenia (SCZ) have a pervasive impairment in the communicative-pragmatic domain^3–11^, characterized by severe difficulties in drawing the inferences necessary to correctly recognize the speaker’s communicative intentions.

Communicative-pragmatic deficits in patients with SCZ have been described at multiple levels: previous studies reported difficulties in the comprehension of non-literal and figurative forms of language, namely indirect speech acts^12,13^, irony^4,6,7,9^, metaphors and idioms^5,8,14,15^, as well as narrative and conversational impairment^16^, and in the recognition and recovery of communicative failures^17^ and deceitful communicative acts^18–20^. Moreover, some studies showed that patients exhibit an increasing level of difficulty in the comprehension and production of communicative acts proffered with different communicative intentions: subjects with SCZ showed a decreasing trend of performance in the comprehension and production of sincere, deceitful, and ironic communicative acts^7,19^. The authors explained this tendency as being due to the increasing inferential ability necessary to correctly comprehend each type of pragmatic expression^7,19^. These studies suggested that communicative difficulties in SCZ might mainly stem from a specific problem in the inference-making processes necessary to derive the speaker’s (correct) communicative intention.

Recently, communicative-pragmatic language dysfunction was proposed as an important cognitive marker for the disorder^10,21–23^, with good prediction accuracy in classifying patients with SCZ vs. controls^24,25^, and as being associated with specific clinical features of the disorder^4,26–28^. In particular, deficits in communicative-pragmatic abilities were associated with clinical traits such as disorganized/concrete symptoms^26^, positive symptoms^29,30^, and formal thought disorders^4^. Moreover, pragmatic dysfunction has been found to be related to the social impairment seen in the disorder, with several studies reporting an association between deficits in Theory of Mind (ToM), i.e., the ability to ascribe mental states to others^31^, and pragmatic impairments^6,9,13,32^. Despite some studies used pragmatic tasks, as irony or sarcasm comprehension, to investigate ToM^33^, recent researches highlighted how pragmatic ability and ToM are two not completely overlapping domains^8,24,34–37^. These research showed as the development of pragmatic competence is supported by a functional maturation of inference-making brain structures rather than a maturation of ToM areas^38–40^, as pragmatic and Theory of Mind deficits are partially dissociated in many clinical conditions^8,9,19,41–44^, and as pragmatic and theory of mind tasks involve partially different neural circuits^37,45–50^, thus suggesting the specificity of communicative ability with respect to other cognitive domains^39,40,42,51^.

While previous studies investigated pragmatic performance of patients with SCZ by using a variety of different pragmatic tasks, little research has focused on analyzing the errors committed in the comprehension of different communicative acts. Error analysis may be highly informative as to the clinical and cognitive processes underlying patients’ failures in communicative-pragmatic tasks and may thus provide a critical insight into the condition and its clinical features. Indeed, different factors can underlie difficulties of patients with SCZ in recognizing different communicative intentions, leading to different error patterns.

Individuals with SCZ have a strong tendency to misattribute intentions to other persons. For example, patients with high degree of suspiciousness may interpret a simple question like “Do you live nearby?”, when asked by a passer-by, as an attempt to force them to reveal personal information in order to deceive or circumvent them. Or they might attribute a communicative intention to a person who has no intention of communicating with them^3,52,53^. This tendency could be related to clinical symptoms, such as persecutory delusion, which may lead patients to attribute malevolent intents to other people, such as those of deceiving or harming them. Indeed, previous studies showed that patients with SCZ have a strong tendency to misattribute the speaker’s communicative intentions, and that this tendency can interact with the condition and its clinical features such as paranoid symptoms^18,29^ and persecutory delusions^30^.

Different response patterns in pragmatic tasks can thus reflect an a priori tendency, i.e., bias, to select a specific response category instead of another, which in turn may reflect specific aspects of the disease and its clinical features. However, by only analyzing patients’ overall pragmatic performance, i.e., accuracy rate, it is difficult to disentangle whether their errors are due to a specific bias in attributing some kind of communicative intention to others, such as a tendency to interpret a statement as deceitful or literal, or to a diffuse, global deficit in interpreting communicative intentions irrespective of the specific kind of communicative act. Instead, by analyzing error patterns it is possible to identify whether a systematic bias characterizes the errors committed by patients with SCZ, and in that case to determine their tendency to respond by selecting a specific response category. Error analysis may thus help to shed light on potential causes of patients’ difficulties in inferring communicative intentions and to evaluate the relationship between these deficits and specific clinical features of the disorder.

Signal detection theory (SDT) is a framework used to model performance in tasks which require participants to identify when a stimulus is present (signal) and when the stimulus is absent (noise)^54–57^. The advantages of using SDT to analyze participants’ performance in pragmatic tasks is that it allows to evaluate separately sensitivity i.e., the ability of participants to correctly identify when the signal is present (e.g., the correct identification of the speaker’s ironic communicative intention) while avoiding incorrect identification (false alarms), and response bias, i.e., an a priori tendency to give one response over another. The latter measure may prove particularly useful in explaining systematic patterns of error committed by participants. Signal‐detection theory may thus provide a useful framework for a more in-depth analysis of participants’ accuracy and a more detailed analyses of participants’ errors patterns. No previous study used the SDT to analyze errors patterns in tasks assessing patients’ with SCZ ability to comprehend another’s person communicative intentions.

The main goal of the present study is to investigate error patterns in patients with SCZ during a task assessing the comprehension of different communicative acts, i.e., sincere, deceitful and ironic communicative acts, and their relationship with the clinical features of SCZ.

The originality of the present study is in the focus on error analysis using the signal detection theory framework. SDT analysis can be used to quantify the ability of participants to correctly detect the signal when it is present, i.e., sensitivity, and their tendency to wrongly perceive the stimulus when it is not present, i.e., response bias, for example the tendency to recognize a statement having a specific communicative intention, such as being ironic even when it is not ironic, but is, instead, sincere or deceitful, for instance. We aim to investigate whether patients with SCZ exhibit a specific a priori tendency, i.e., bias, to select a specific response category instead of others. Furthermore, we wish to investigate the relationship between sensitivity and response bias and specific clinical features of the disorder such as: symptom severity, pharmacological treatment, and personal and social functioning.

## Results

### Demographic and clinical characteristics of the sample

Demographic characteristics of patients with SCZ and HC are reported in Table 1. No statistically significant differences emerged between the two groups in age, education, and gender (Two-sided *T*-tests: 0.90 > *t* > 0.50, 0.82 > *p* > 0.75, see Table 1). Clinical characteristics of the SCZ group are shown in Table 2.

**Table 1:** Demographic characteristics.

	SCZ group	HC group	Statistic *F*/χ^2^	*p* value
Age, years	40.2 (11.7)	39.4 (11.5)	0.050	0.824
Gender (M/F)	16/8	17/7	0.097	0.755
Education, years	13,7 (4,3)	13,9 (4,2)	0.090	0.765

*M* male, *F* female, *SCZ* schizophrenia, *HC* healthy control, Mean and standard deviation (SD).

**Table 2:** Clinical characteristics.

Age at illness onset, years	26.50 (7.63)
Duration of illness, years	14.16 (10.33)
PANSS – POS, score	7.42 (2.02)
PANSS – DIS, score	6.96 (2.61)
BNSS – Avl, score	21.83 (8.28)
BNSS – ExD, score	12.00 (6.65)
CDSS, total score	3.88 (4.90)
PSP, score	60.29 (13.94)
CPZ equivalent, mg/day	390.22 (144.62)

*PANSS* Positive and Negative Syndrome Scale, *POS* positive symptoms, *DIS* disorganized/concrete symptoms, *BNSS* Brief Negative Symptoms, *Avl* avolition dimension, *ExD* expressive deficit dimension, *CDSS* Calgary Depression Scale for Schizophrenia, *PSP* Personal and Social Performance Scale, *CPZ* chlorpromazine. Mean and standard deviation (SD).

### Descriptive statistics of the pragmatic task

The mean accuracy rate (SD) of control participants was 93.8 (10.8) for the sincere condition, 82.3 (13.4) for the deceitful condition, and 83.7 (13.3) for the ironic condition; the mean accuracy rate of participants with SCZ was 81.3 (19.2) for the sincere condition, 66.31 (27.5) for the deceitful condition, and 57.0 (24.5) for the ironic condition (see Table 3).

**Table 3:** Descriptive statistics of participants’ performance in the pragmatic task.

	Communicative acts					
Measures	Sincere		Deceit		Irony	
	SCZ	HC	SCZ	HC	SCZ	HC
Hits/accuracy	9.75 (2.31)	11.25 (1.29)	7.96 (3.3)	9.88 (1.60)	6.84 (2.94)	10.02 (2.27)
False alarms	2.3 (3.24)	1.0 (1.59)	4.17 (2.85)	1.71 (2.22)	2.80 (2.50)	1.54 (1.38)
Responses not given	0.63 (0.92)	0.21 (0.41)	0.79 (1.14)	0.21 (0.41)	0.88 (1.03)	0.17 (0.38)
Correct rejections	21.75 (3.3)	23 (1.59)	19.92 (2.80)	22.3 (2.22)	21.29 (2.53)	22.42 (1.38)

For each subject we report the mean number and standard deviation of hits (correct responses), false alarms (incorrect responses), responses not given, and correct rejections for each category (i.e., sincere, deceitful, and ironic communicative acts) in the two groups (SCZ and HC).

### Signal detection analysis

**Fig. 1:**
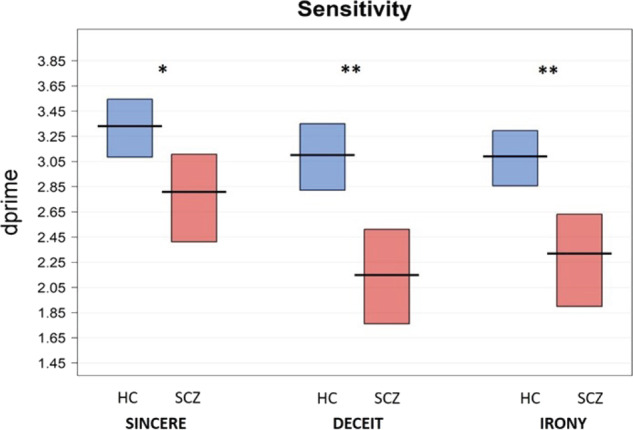
Sensitivity (d-prime) values for sincere, deceitful, and ironic communicative acts in the SCZ and HC groups. Note: The horizontal line corresponds to the mean of the different groups (SCZ = schizophrenia, HC = healthy controls) for the different communicative acts (Sincere: SCZ = 2.81 (sd = 0.81), HC = 3.33 (0.56), Cohen’s *d*: 0.75 [0.16, 1.34]; Deceit: SCZ = 2.15 (0.88), Cohen’s *d*: 1.24 [0.62, 1.86]; HC = 3.01 (0.63); Irony: SCZ = 2.32 (0.87), HC = 3.09 (0.48), Cohen’s *d*: 1.10 [0.48, 1.70]), and the rectangle represents the Bayesian highest density interval. **p < 0.01 *, *p* < 0.05.

The results showed a significant effect of *Group* on sensitivity (d’), i.e., the probability of participants giving correct responses (hits) for each category of communicative acts while avoiding incorrect ones (false alarms). Sensitivity provides a measure of how a participant is able to detect the signal (correct responses) outside of subjective bias (false alarms), while accuracy, usually used in traditional behavior analysis, only quantifies correct responses. In detail, patients showed lower sensitivity than controls, that is, they were not as good at identifying the speaker’s communicative intention (signals) for all the communicative phenomena examined, i.e., sincere (*b* = −0.41, SE = 0.19, *t* = −2.17, *p* < 0.05), deceitful (*b* = −0.86, SE = 0.19, *t* = −4.51, *p* < 0.0001) and ironic communicative acts (*b* = −0.67, SE = 0.19, *t* = −3.54, *p* < 0.001). Participants with SCZ were less able than controls in identifying the speaker’s communicative intention (signals) in correct trials (hits, e.g., irony in ironic trials) while avoiding incorrect responses (false alarms, e.g., responding irony in non-ironic trials). The analysis also showed a significant effect of general intelligence (*b* = 0.27, SE = 0.07, *t* = 3.71, *p* < 0.001), i.e., a higher level of general intelligence was associated with higher sensitivity (see Table 4 and Fig. 1). However, differences in sensitivity between patients and controls for the different communicative acts still remained significant after controlling for the role of the covariates, i.e., level of intelligence, age, and gender.

**Table 4:** Regression models comparing patients with SCZ and HC.

Model specification	Fixed effects	*b*	SE	*t*	*p*-value
Sensitivity					
dprime ~ Communicative phenomenon + Group + Age + Gender + Level of intelligence + (1 | ID)	Sincere	3.15	0.14	22.75	<0.001
	Deceit	2.91	0.14	21.01	<0.001
	Irony	2.93	0.14	21.18	<0.001
	Level of intelligence	0.27	0.07	3.71	<0.001
	Age	−0.14	0.07	−1.97	0.055
	Gender	−0.29	0.16	1.82	0.076
	Sincere: Group = SCZ	−0.41	0.19	−2.17	<0.05
	Deceit: Group = SCZ	−0.86	0.19	−4.51	<0.001
	Irony: Group = SCZ	−0.67	0.19	−3.54	<0.001
Response bias					
Beta values ~ Communicative phenomenon + Group + Age + Gender + Level of intelligence + (1 | ID)	Sincere	0.78	0.11	6.82	<0.001
	Deceit	0.83	0.12	6.93	<0.001
	Irony	0.89	0.12	7.04	<0.001
	Level of intelligence	0.02	0.06	0.29	0.76
	Age	0.01	0.01	0.09	0.93
	Gender	−0.11	0.12	−0.94	0.35
	Sincere: Group = SCZ	−0.05	0.14	−0.35	0.72
	Deceit: Group = SCZ	0.40	0.19	2.07	<0.05
	Irony: Group = SCZ	−0.19	0.15	−1.29	0.20

*b* beta regression coefficient, *SE* standard error, *t* *t*-value. The response bias model was fitted using a Gamma distribution with an inverse link, thus positive beta regression coefficients (e.g., 0.40 for deceit in group of patients with SCZ) indicate lower beta values.

Response bias represents the extent to which participants tend to commit errors by choosing a specific response category. This makes it possible to identify a specific error tendency which may be informative as to the clinical and cognitive factors underlying patients’ failures.

**Fig. 2:**
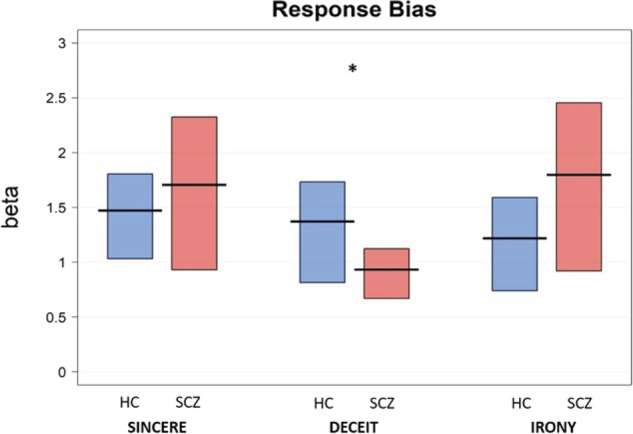
Response bias (Beta) values for sincere, deceitful, and ironic communicative acts in SCZ and HC groups. Note: The horizontal line corresponds to the mean of the different groups (SCZ = schizophrenia, HC = healthy controls) for the different communicative acts (Sincere: SCZ = 1.71 (sd = 1.63), HC = 1.47 (0.96), Cohen’s *d*: −0.17 [−0.74, 0.39]; Deceit: SCZ = 0.93 (0.55); HC = 1.37 (1.06), Cohen’s *d*: 0.52 [−0.06, 1.09]; Irony: SCZ = 1.80 (1.77), HC = 1.22 (1.00), Cohen’s *d*: −0.40 [−0.97, 0.17]), and the rectangle represents the Bayesian highest density interval. Higher Beta values indicate more conservative responding, while lower Beta values indicate more liberal responding. **p < 0.01 *p < 0.05.

We found a significant effect of *Group* on response bias, as participants with SCZ showed a lower Beta value for deceitful communicative acts when compared to healthy controls (*b* = 0.40, SE = 0.19, *p* < .05). A lower Beta value corresponds to a stronger response bias and a more liberal response criterion, i.e., a lower threshold, compared to HC, for choosing the deceitful category as a response option. In other words, patients with SCZ showed a greater tendency to respond “deceit” in both deceitful and non-deceitful trials than controls (see Table 4 and Fig. 2). We found no differences between patients and controls on response bias for sincere (*b* = −0.05, SE = 0.14, *p* = 0.72) and ironic communicative acts (*b* = −0.19, SE = 0.15, *p* = 0.20). We found no effect of covariates, i.e., general intelligence, age, and gender on response bias.

### Relationship with clinical variables

In the group of participants with SCZ, the regression analysis showed an effect of disorganized/concrete (DIS) symptoms on sensitivity: patients with higher DIS symptoms rated using the PANSS showed a lower sensitivity for irony (*b* = −0.49, SE = 0.16, *p* < 0.01). Severity of positive (POS), negative (Avl and ExD factors), and depressive (CDSS score) symptoms, and antipsychotic daily equivalent dosage (CPZ equivalent), were not significantly associated with sensitivity. Response bias was not significantly affected by any of the clinical variables assessed (see Table 5). Finally, we did not find a significant role of beta and *d*’ values in predicting patients’ performance on the PSP scale.

**Table 5:** Selected models for the clinical factors predicting sensitivity and response bias in the SCZ group.

Model specification	Fixed effects	*b*	SE	*T*	*p*-value
Sensitivity					
dprime ~ Communicative phenomenon + Communicative phenomenon: Symptoms = DIS + (1 | I D)	Sincere	2.81	0.16	17.5	<0.001
	Deceit	2.15	0.16	13.3	<0.001
	Irony	2.32	0.16	14.4	<0.001
	Sincere: Symptoms = DIS	−0.32	0.16	−1.98	0.06
	Deceit: Symptoms = DIS	−0.25	0.16	−1.5	0.13
	Irony: Symptoms = DIS	0.49	0.16	−3.0	<0.01
Response bias					
beta ~ Communicative phenomenon + Communicative phenomenon: Symptoms (CDSS) + Communicative phenomenon: Symptoms = Avl + (1 | ID)	Sincere	0.79	0.11	6.95	<0.001
	Deceit	1.15	0.15	7.93	<0.001
	Irony	0.74	0.11	6.78	<0.001
	Sincere: Symptoms = CDSS	−0.01	0.10	−0.13	0.89
	Deceit: Symptoms = CDSS	−0.7	0.18	−0.39	0.69
	Irony: Symptoms = CDSS	0.27	0.15	1.82	0.07
	Sincere: Symptoms = Avl	−0.26	0.14	−1.89	0.60
	Deceit: Symptoms = Avl	0.13	0.16	0.87	0.38
	Irony: Symptoms = Avl	−0.003	0.1	−0.3	0.98

*b* beta regression coefficient, *SE* standard error, *t t*-value, *ID* participant, *DIS* disorganized/concrete symptoms, *Avl* avolition dimension, consisting of anhedonia, asociality and avolition symptoms of the Brief Negative Symptoms Scale (BNSS), CDSS Calgary Depression Scale for Schizophrenia total score.

## Discussion

In the present study we examined the error patterns of patients with SCZ and healthy controls in a task assessing pragmatic comprehension of communicative acts uttered with different communicative intentions, i.e., sincere, deceitful, and ironic. More specifically, we set out to investigate whether patients with SCZ exhibit specific a priori tendencies, i.e., bias, to select a specific response category, and to shed light on the relationship between such tendencies and the clinical features of the disorder. To this aim we used the signal detection theory framework to model participants’ performance and quantify their ability to discriminate between signal and noise, i.e., sensitivity, and their tendency to respond by choosing a specific response category, i.e., response bias.

The results confirmed that the ability to infer the speaker’s communicative intention is impaired in SCZ. More specifically, in line with previous studies^5–9,58^, we found that patients with SCZ exhibited lower sensitivity, in terms of the ability to correctly detect the speaker’s communicative intentions, compared to healthy controls, for all the pragmatic phenomena evaluated, i.e., sincere, deceitful, and ironic communicative acts. The sensitivity measure has some advantages over traditional accuracy measures, which only quantify correct responses. Indeed, by using this measure, we showed that the difficulties of patients in recognizing the speaker’s communicative intentions, i.e., lower sensitivity, are due to different reasons. In the case of irony, such lower sensitivity is mainly due to a low proportion of correct responses in ironic trials. Instead, in the case of deceit, patients showed a higher proportion of correct responses than for irony, but also a higher rate of false alarms, so that sensitivity values were similar. We also found a significant role of general intelligence in explaining the sensitivity measure, which confirms previous evidence showing that intelligence may have a role in pragmatic understanding^59,60^. However, even after controlling for the role of general intelligence, the differences in the sensitivity index between patients and controls remain significant, pointing to the specificity of the deficit in the recognition of communicative intentions^5,6,61^. Overall, this result supports recent studies proposing pragmatic impairment as a core feature of SCZ^10,21–25^, with patients exhibiting severe deficits in high-level language skills.

Further, by analyzing participants’ error patterns, we found that, when committing errors, patients with SCZ tend to select the “deceitful” category more often than the other ones, i.e., sincere and ironic. This is reflected in a stronger response bias for deceitful communicative acts in the group of patients with SCZ, compared to HC, and indicates a strong a priori tendency of patients to respond “deceit” in both deceitful and non-deceitful trials.

The tendency to misattribute deceitful communicative intentions to other people’s utterances, irrespective of the context and of the true communicative intention, can be explained by the attributional bias characterizing this disorder. This bias describes the way in which individuals explain the causes, or make sense, of social events or interactions^62,63^. Indeed, hostile attributional style is a core dimension of altered social cognition in SCZ^64,65^.

We did not find any relationship between response bias and clinical features of the disorder. This can be explained by the fact that patients were clinically stable and achieved low or moderate scores in all the psychopathological dimensions assessed. Our result is consistent with previous studies which demonstrated that one-sided and monocausal attribution styles, sustained by poor metacognition, are trait markers of SCZ, independently of the severity of symptoms^66,67^. In this view, the impairment in metacognitive monitoring on cognitive tasks combined with a hostile attributional bias can explain patients’ response bias, i.e., a more liberal response tendency, for “deceit” observed in the present study without needing the involvement of specific symptoms.

This finding is in line with previous studies showing that patients with SCZ tend to misattribute other people’s intentions. However, previous studies mainly focused on mentalizing errors, such as erroneous attribution of beliefs, emotions, and desires, and found that these errors may concur with the maintenance of clinical symptoms, such as persecutory delusions^68,69^. In the present study, we rather focused on investigating the recognition of communicative intentions in SCZ during a communicative-pragmatic task. We showed that the tendency to misattribute mental states also extends to the ascription of communicative intentions, specifically resulting in a bias which leads patients to misattribute deceitful communicative intentions.

As far as the relationship between sensitivity and clinical features is concerned, we found that the sensitivity measure for the irony condition was related to disorganized/concrete symptoms, i.e., conceptual disorganization (P2), difficulty in abstract thinking (N5), and poor attention (G11) of the PANSS. In detail, these three psychopathological elements were associated with a low level of accuracy in the irony condition principally due to a small proportion of correct responses in ironic trials and not to a wrong choice of this communicative intention in non-ironic trials (i.e., sincere and deceitful). In other words, the more severe the disorganized/concrete symptoms, the less patients choose irony as an option. This result is consistent with other studies on irony detection where people with SZ were asked to recognize irony in a pragmatic task^19,70^. Furthermore, the association between the severity of disorganized/concrete symptoms and the difficulty in detecting irony is in line with a previous study^26^ that reported a significant relationship between higher scores in the PANSS items conceptual disorganization (P2) and difficulty in abstract thinking (N5) and concretism, i.e., the tendency to adhere to the literal meaning of utterances. In this view, the impaired comprehension of both ironic communicative acts and metaphors and proverbs is probably sustained by a common inferential deficit closely linked to disorganized/concrete symptoms.

The present study demonstrated the potential of using signal detection analysis in modeling error patterns of patients with SCZ in a communicative-pragmatics task. For example, previous studies observed that patients with SCZ have a strong tendency to adhere to the literal meaning of an utterance, i.e., concretism, and that this tendency may be responsible for patients’ errors in comprehending figurative or non-literal expressions. Patients may be biased toward the literal interpretation of an utterance, thus overlooking the non-literal and indirect meaning^27,42,71^. A recent study by Bambini et al.^26^, investigated concretism in SCZ across different figurative expressions, i.e., proverbs, idioms and metaphors, and pragmatic tasks. The authors found figurative language comprehension to be largely impaired in SCZ, and observed that the tendency for concretism is linked to the clinical features of the disease, i.e., the presence of formal thought disorder and difficulties in abstract thinking. A future avenue of investigation would be to test whether patients with SCZ show a response bias toward the literal interpretation of figurative expressions using the signal detection theory framework. We did not observe this tendency in the present study, although we were not specifically concerned with evaluating the comprehension of figurative expressions for which concretism has been reported in previous studies^5,72,73^.

A limit of the present study regards the characteristics of our sample. Indeed, we tested a population of patients with a chronic and stable disease, while it would be interesting to assess whether the observed bias differs for patients with a short disease history (<2 years). Finally, the sample size of this study is relatively small. Further studies are needed to replicate the present findings and further elucidate the relationship between symptoms, neuro and social cognition, and pragmatic comprehension in people with SCZ.

In conclusion, we systematically analyzed error patterns in patients with SCZ and healthy controls (HC) during a task assessing the comprehension of different communicative acts, and their relationship with the clinical features of SCZ. We found patients with SCZ to be impaired in their ability to infer the speaker’s communicative intention for all the pragmatic phenomena evaluated, i.e., sincere, deceitful and ironic communicative acts, and that the impairment in irony recognition is related to disorganized/concrete symptoms. Moreover, patients with SCZ showed a stronger response bias for deceitful communicative acts, possibly related to the attributional bias characterizing the disorder. The study showcases the potential of modeling error patterns of patients with SCZ during a communicative-pragmatics task and offers useful suggestions for the creation of specific rehabilitation programs^74,75^ focused on helping patients to overcome their communicative difficulties. A correct comprehension of other people’s communicative intentions is essential to achieve real-life goals like getting and maintaining a job and having stable interpersonal relationships. Therefore, the implementation of neurocognitive rehabilitation and social skills training combined with treatments^74,75^ specifically focused on communicative-pragmatic ability could broad the spectrum of activity of the cognitive remediation programs, thus enhancing their efficacy in improving interpersonal and work functioning of people with SZ.

## Methods

### Participant

Twenty-four individuals with established SCZ and 24 healthy controls (HC) were included in the study. Patients with SCZ and HC were matched for age, gender, and education. Individuals diagnosed with SCZ met the following criteria: (1) diagnosed with schizophrenia according to DSM 5 (APA 2013) criteria, confirmed using the SCID 5 CV (APA 2015), (2) no other current diagnosis of mental disorder other than schizophrenia, (3) clinically stable, i.e., absence of hospitalization and treatment modification in the last six months. All participants had to meet the following inclusion criteria to take part in the experiment: (1) aged between 18 and 65 years, (2) no previous history of neurological illness, (3) basic cognitive and linguistic abilities demonstrated by achieving a cut-off score in the following neuropsychological tests: Test di Intelligenza Breve^76^, Italian equivalent of the National Adult Reading Test^77^ (NART; cut-off score 70) and two sub-scales (Comprehension of written words and comprehension of written sentences) of the Aachener Aphasie Test^78^ (AAT; cut-off 112/120), (4) Italian native speakers. HC had to meet the following inclusion criteria: (1) no current use of psychoactive drugs, (2) no personal or family history of psychiatric disorders. All participants gave their written informed consent and took part in the study on a voluntary basis. The study was approved by the Local Research Ethics Committee (protocol number: 0076364).

### Clinical assessment

The Positive and Negative Syndrome Scale (PANSS)^79^ was used to rate the severity of positive (POS) and disorganized/concrete symptoms (DIS), according to the solution proposed by Wallwork et al.^80^. Positive symptoms were assessed using four items of the PANSS: P1 (delusions), P3 (hallucinatory behavior), P5 (grandiosity), G9 (unusual thought content). Disorganized/concrete symptoms were assessed using three items of the PANSS scale: P2 (conceptual disorganization), N5 (difficulty in abstract thinking), and G11 (poor attention). Negative symptoms were assessed using the Italian version of the Brief Negative Symptoms Scale (BNSS)^81^, and grouped into two factors: “avolition” (Avl), consisting of anhedonia, asociality and avolition, and “expressive deficits” (ExD), including blunted affect and alogia^82,83^. Depressive symptoms were evaluated using the Calgary Depression Scale for Schizophrenia (CDSS)^84^. Higher scores represent greater symptom severity. The level of functioning was evaluated with the Personal and Social Performance Scale^85^ (PSP). Higher scores represent a higher level of functioning. Antipsychotic dosage was converted to chlorpromazine (CPZ) equivalent dose using an established conversion methodology^86^. The clinical characteristics of the SZ group are summarized in Table 2.

### Assessment of general intelligence

To estimate general intelligence, we used “Test di Intelligenza Breve (TIB, i.e., Brief Intelligence Test^76^), an Italian version of the National Adult Reading Test (NART). The TIB consists of a list of 54 words (34 words with irregular accent for the actual test and 20 words with high frequency of use as control stimuli), which the participants have to read aloud. The final score is calculated by summing up the number of words pronounced correctly. The IQ score (total IQ) is then estimated by using the formula reported in Colombo et al.^76^.

### Pragmatic assessment

The assessment was conducted using 36 short stories, each followed by a target sentence designed to test participants’ comprehension of sincere, deceitful, and ironic communicative acts. Each story was made up of two parts, i.e., a context and a target sentence. The first part explained the context and outlined the scenario in which the events would take place. Each story had two characters and ended with one of them saying something to the other, whose answer was the target sentence. We used three different context-scenarios to propose three different communicative intentions: sincere, deceitful, and ironic (see Supplementary Table 1 for an example of the story). The three context-scenarios associated with each (identical) target sentence were comparable in their level of difficulty, number of words and syllables, and the Gulpease readability index (see Supplementary Table 2). The experimental material had already been validated in a previous study to make sure the target sentences were correctly interpreted (see Bosco et al.^49^).

### Procedure

At the beginning of the session, subjects were tested using the ‘Test di Intelligenza Breve’ (TIB) and the other cut-off tests, i.e., the two sub-scales (Comprehension of written words and comprehension of written sentences) of the Aachener Aphasie Test (AAT). Next, participants completed the communicative-pragmatic task, during which they read the story contexts followed by each target sentence. When the target sentence disappeared, participants had to recognize the speaker’s communicative intention by pressing a button to choose from among the three alternative response options: (1) sincere (2) deceitful (3) ironic. Correct responses received a score of 1. We created two different protocols (A and B) so that the order of trial presentation was pseudorandomized and counterbalanced across participants. Participants completed the task individually. When they arrived at the hospital, they were instructed on the task and they completed three practice trials (not included in the final stories) before the start of the experiment. The experimental task was divided into two blocks each lasting approximately 20 min, with a brief pause (2–3 min) between the two blocks, for a total time of 45 min.

### Statistical analysis

Statistical analyses were performed using R software and Psycho package^87,88^. First we performed the signal detection analysis. The responses given in the pragmatic task used in the present study were analyzed using the signal detection theory (SDT) framework. SDT is generally applied when participants have to identify two (or more) different stimuli, where the task consists in discerning when the stimulus is present (signal) and when the stimulus is absent (noise). In our task, the correct identification of the speaker’s communicative intention corresponds to a hit, i.e., the probability of the subject reporting the signal as present when it actually is present. Instead, the incorrect identification of the speaker’s intention (e.g., interpreting an ironic statement as deceitful or sincere) corresponds to a false alarm, i.e., the probability of the subject reporting that the signal is present when it is absent. SDT analysis models the relationship between hit and false alarm rates and provides distinct quantitative indices of: a) *Sensitivity*, which is the ability to discriminate between signal and noise, corresponding in our task to the ability of a subject to correctly identify the speaker’s communicative intention. Sensitivity is calculated as the standardized value of the hit rate (i.e., correct responses) minus giving correct responses (hits) for each category while avoiding incorrect ones (false alarms). In this way, it offers more advantages than a simple measure of accuracy, which only quantifies b) *Response bias*, which is the general tendency of a subject to respond by choosing a specific response category (e.g., deceit). For example, in our task a participant may have a tendency to respond “deceit”, and thus be more likely to respond “deceit” regardless of the type of trial (i.e., sincere, deceitful or ironic). This will result in a high proportion of “deceit” responses in signal trials (i.e., deceit trials, when the communicative intention was deceitful), and thus in a high hit rate (correct responses for the deceitful condition). However, it will also result in high rate of “deceit” responses in noise trials (i.e., sincere or ironic conditions), and thus in a high rate of false alarms. In this case, we say the subject is using a *liberal criterion*, meaning the criterion (threshold) s/he is adopting to decide whether a statement is deceitful or not is very low, and this results in a high proportion of “deceit” responses in both deceitful and non-deceitful (sincere and ironic) trials. The opposite situation occurs when a subject has a tendency to not respond “deceit”. This will result in a low proportion of “deceit” responses in signal trials (i.e., deceit trials), and thus in a lowhit rate (correct responses); but it will also result in a low rate of “deceit” responses in noise trials (i.e., sincere or ironic trials), and thus in a low rate of false alarms. In this latter case the subject isusing *a conservative criterion*, meaning the criterion (threshold) s/he is adopting to decide whether a statement is deceitful or not is very high, and this results in a low proportion of “deceit” responses in both deceitful and non-deceitful trials. The advantages of using SDT to analyze participants’ performance in pragmatic tasks is that it allows *sensitivity* i.e., the ability of participants to correctly identify the speaker’s communicative intention (i.e., signal) while avoiding incorrect identification (false alarms), and *response bias*, i.e., an a priori tendency to give one response over another, to be evaluated separately. The latter measure may prove particularly useful in explaining systematic patterns of error committed by participants. For each subject we computed the number of hits (correct responses), false alarms (incorrect responses, e.g., interpreting an ironic statement as deceitful), responses not given, and correct rejections (not responding “irony” when irony is not present, as in deceitful and sincere trials) for each category (i.e., sincere, deceitful, and ironic communicative acts). As an example, if a participant correctly recognizes 8/12 ironic trials, and wrongly recognizes 4 non-ironic trials as ironic, this will result in 8/12 hits and 4/24 false alarms for the irony category. We then computed measures of sensitivity and response bias (B) using the R package Psycho software according to the formula provided in Pallier^89^. When the bias toward one response over another increases, i.e., the subject uses a liberal criterion, beta values decrease to 0. By contrast, when the bias toward one response over another decreases, i.e., a subject is using a conservative criterion, beta values increase to more than 1 on an open-ended scale. Then a generalized linear model (GLM) was employed in order to analyze the differences between patients with SCZ and HC. The GLM was set with sensitivity (d’) and response bias (B) indices as the respective outcome, diagnosis (Groups: SCZ and HC) and type of phenomenon (sincere, deceit and irony) as fixed factors (separately evaluating the effect of Group by type of phenomenon), age, and general intelligence as continuous covariates, gender as categorical covariate, and varying effects by participant^90,91^. Scores of participants in the communicative-pragmatic tasks and *d*-prime values (sensitivity) were normally distributed in the two groups (HC and SCZ), as indicated by the Kolmogorov–Smirnov test of normality (HC: D(24) = 0.143, *p* = 0.20; SCZ: D(24) = 0.132; *p* = 0.20). Distribution of beta values is, by definition, left-bounded and tends to be positively skewed; we therefore used Gamma distribution which best approximates the distribution of response bias. For a summary of the model see Table 4. Subsequently, to analyze the role of the clinical variables on sensitivity and response bias, we used a GLM with sensitivity (d’) and response bias (B) indices as outcome, separately evaluating for each communicative phenomenon (sincere, deceit, and irony) the effect of clinical variables as predictors, and varying effects by participants. We included relevant predictors, i.e., severity of disorganized (DIS), positive (POS), negative (Avl and ExD factors), depressive (CDSS score) symptoms, and antipsychotic daily equivalent dosage (CPZ equivalent) in the model starting from a null model including only intercept, and then checking at each step whether the addition of each factor corresponds to a significant increase in goodness of fit using the likelihood ratio test and the Akaike information criterion (AIC). The final models are reported in Table 5. Finally, to analyze the role of sensitivity and response bias values on the social and personal functioning (PSP score), we used a generalized linear model with PSP score as outcome, and evaluating the effect of beta and *d*-prime values for each pragmatic phenomenon (sincere, deceit, and irony) as predictors. We adopted the same procedure detailed above for the inclusion of relevant predictors in the model.

## Supplementary information

Supplementary information

Related Manuscript File

## Data Availability

Due to the anonymity guaranteed in the informed consent paperwork at the time when data were collected, data cannot be publicly shared, and are controlled by the Comitato Etico Interaziendale of the A.O.U. Città della Salute e della Scienza di Torino. Researchers who wish to request access to these data may contact direzione.psicologia@unito.it (Department of Psychology, University of Turin, Italy) or the corresponding author.

## Code availability

The code of the analysis is available upon request to the first author (alparola@unito.it).
