# Author Correction: Understanding communicative intentions in schizophrenia using an error analysis approach

**DOI:** 10.1038/s41537-021-00152-5

**Published:** 2021-04-30

**Authors:** Alberto Parola, Claudio Brasso, Rosalba Morese, Paola Rocca, Francesca M. Bosco

**Affiliations:** 1 Dipartimento di Psicologia, Università degli Studi di Torino, Torino, Italia; 2 Dipartimento di Neuroscienze “Rita Levi Montalcini”, Università degli Studi di Torino, Torino, Italia; 3 Institute of Public Health, Faculty of Biomedical Sciences, Università della Svizzera italiana, Lugano, Switzerland; 4 Faculty of Communication, Culture and Society, Università della Svizzera italiana, Lugano, Switzerland

**Keywords:** Schizophrenia, Human behaviour

Correction to: *npj Schizophrenia* 10.1038/s41537-021-00142-7, published online 26 February 2021

The original version of this Article contained an error in the spelling of the author Alberto Parola, which was incorrectly given as Parola Alberto. This has now been corrected in both the PDF and HTML versions of the Article.

