# Supplementary material for: Understanding communicative intentions in schizophrenia using an error analysis approach
Source: NPJ Schizophr. 2021 Feb 26;7:12. doi: 10.1038/s41537-021-00142-7 (PMC7910544; doi:10.1038/s41537-021-00142-7)
Supplement: Supplementary file 1 — Supplementary information [file 41537_2021_142_MOESM1_ESM.pdf]

Supplementary Information

Supplementary Table 1. *Example of experimental stories (sincere, deceitful and ironic) associated with the same identical target sentence used in the pragmatic task*

AREA OF INTEREST (AI)	ORIGINAL ITALIAN	ENGLISH TRANSLATION
Context - Sincere	Marco e Luca stanno facendo i compiti di matematica e sono alle prese con un problema molto complesso. Marco non sa come andare avanti. Luca scopre la soluzione con un'intuizione e chiede conferma a Marco. Marco risponde:	Marco and Luca are doing their math homework and are struggling with a very difficult problem. Marco doesn't know how to continue. Luca finds the solution by intuition and asks Marco to confirm it. Marco replies:
Context - Deceitful	Andrea è invidioso di Mario perché a scuola è più bravo di lui. Mario ripete la lezione su cui saranno interrogati il giorno dopo ad Andrea ma commette numerosi errori. Mario pensa di aver fatto bene e chiede conferma ad Andrea. Andrea risponde:	Andrea envies Mario because he does better at school than him. Mario helps Andrea revise the lesson on which they will be tested the next day but makes lots of mistakes. Mario thinks he has done well and asks Andrea for confirmation. Andrea replies:
Context - Ironic	Giovanni ed Alfredo stanno giocando a pallone in giardino. Giovanni calcia la palla con molta forza e colpisce un vaso poggiato sopra il davanzale. Il vaso cade a terra rompendosi. Giovanni guarda Alfredo perplesso. Alfredo risponde:	Giovanni and Alfredo are playing ball in the garden. Giovanni kicks the ball very hard and hits a plant pot standing on a windowsill. The pot falls to the ground and breaks. Giovanni looks at Alfredo with a puzzled expression. Alfredo replies:
TARGET ANSWER (Identical all the versions)	**"Complimenti, ben fatto"**	**"Well done!"**

Supplementary Table 2. *The mean number of words, syllables and the Gulpease index for target sentence and the three different contexts (sincere, deceitful, ironic) associated with each target sentence*

Condition	Mean Length (SD) - number of words	Mean Length (SD) – number of syllabes	Mean Gulpease (SD)
Sincere Context	44.1 (4.3)	91.5 (7.8)	67.8 (5.1)
Deceitful Context	46.3 (4.0)	89.7 (8.4)	71.7 (4.5)
Ironic Context	44.5 (5.5)	91.0 (9.6)	72.2 (.5.9)
Target Sentence	5.4 (1.8)	10.9 (2.2)	94.5 (6.6)
